# Analysis of Features of Alzheimer’s Disease: Detection of Early Stage from Functional Brain Changes in Magnetic Resonance Images Using a Finetuned ResNet18 Network

**DOI:** 10.3390/diagnostics11061071

**Published:** 2021-06-10

**Authors:** Modupe Odusami, Rytis Maskeliūnas, Robertas Damaševičius, Tomas Krilavičius

**Affiliations:** 1 Department of Multimedia Engineering, Kaunas University of Technology, 44249 Kaunas, Lithuania; modupe.odusami@ktu.edu (M.O.); rytis.maskeliunas@ktu.lt (R.M.); 2 Department of Applied Informatics, Vytautas Magnus University, 44248 Kaunas, Lithuania; tomas.krilavicius@vdu.lt

**Keywords:** Alzheimer disease, mild cognitive impairment, magnetic resonance imaging, deep learning, residual neural network

## Abstract

One of the first signs of Alzheimer’s disease (AD) is mild cognitive impairment (MCI), in which there are small variants of brain changes among the intermediate stages. Although there has been an increase in research into the diagnosis of AD in its early levels of developments lately, brain changes, and their complexity for functional magnetic resonance imaging (fMRI), makes early detection of AD difficult. This paper proposes a deep learning-based method that can predict MCI, early MCI (EMCI), late MCI (LMCI), and AD. The Alzheimer’s Disease Neuroimaging Initiative (ADNI) fMRI dataset consisting of 138 subjects was used for evaluation. The finetuned ResNet18 network achieved a classification accuracy of 99.99%, 99.95%, and 99.95% on EMCI vs. AD, LMCI vs. AD, and MCI vs. EMCI classification scenarios, respectively. The proposed model performed better than other known models in terms of accuracy, sensitivity, and specificity.

## 1. Introduction

Alzheimer’s disease (AD) features can be analyzed to create more effective and accurate tools based on recent economical, and publicly available, technologies. Currently, there have been several approaches which can be applied to detect AD in its early phases, such as neuroimaging techniques [1,2,3], behavior and emotion analysis [4,5], often referred to as cognitive approaches, and cognitive test. Behavioral analysis methods help to detect irregular reactions to frequent problems in daily living activities, and some of which involve the installation of sensors in the patient’s house. One of the main drawbacks of this strategy is that it comes with a lot of limitations, as it needs the patient’s permission to mount the sensors in his/her home. One of the signs of AD is a decline in social cognition, and some studies have focused on patients’ ability to interpret emotions using various data, such as eye-tracking data [6], voice/speech recordings [7], facial expressions [8], and electroencephalograms (EEG) [9,10].

Neuroimaging techniques such as structural magnetic resonance imaging (sMRI) [11,12], fMRI [13], fluorodeoxyglucose positron emission tomography (FDG-PET) imaging [14], amyloid PET [1], and diffusion tensor imaging (DTI) [15]. These neuroimaging techniques have shown to be promising modalities to assess abnormal brain changes linked to AD, and they remain mainly used in the more advanced centers. In amyloid PET, diffuse amyloid deposits in the cortex are considered a measure of neurodegeneration, and a marker that binds to the Aβ protein is injected into the subject. Amyloid PET shows both quantitative information, that can be regionally based, and qualitative information about the topology of Aβ deposition in the brain. For fMRI, the alteration in blood flow and blood oxygen concentration measurement shows the brain’s metabolic activities [16]. The amount of shrinkage in brain sub-regions, especially the hippocampus, corroborates the structural changes of the brain [16]. FDG-PET provides quantitative measurements of the brain’s metabolic activity [17].

Compared to other neuroimaging modalities, fMRI has helped AD analysts to assess functionally activated regions when conducting a task to diagnose AD early [18]. When performing tasks, the frontal-subcortical-parietal regions, thalamus, striatum, and intraparietal cortex are all co-activated brain regions. Preliminary fMRI studies have shown a strong link between cognitive functions and sensorimotor eye movements, assisting in the development of a better understanding of neurodegenerative diseases’ network-level brain disruptions. Authors in [19] investigated functional brain connectivity, and they concluded that new approaches are needed to comprehend the functional pattern alternation in the initial phase of AD.

The AD functional brain changes among mild cognitive impairment (MCI) stages are closely related and constant trait information from the features that represent each stage are often very complex to delineate. Based on this, it is difficult to accurately predict AD early. Distinguishing the functional brain pattern of MCI stages need accurate information and knowledge. In this paper, to diagnose AD early, authors created a DL algorithm to take out useful features from hippocampal fMRI data from the ADNI database. A clinician may use the proposed model to easily diagnose a patient with MCI and monitor their progress over time.

The contribution of this paper are as follows:

1. This study proposes a modified ResNet18 and performs binary classification of AD which include EMCI/LMCI, AD/CN, CN/EMCI, CN/LMCI, EMCI/AD, LMCI/AD, and MCI/EMCI.
2. To effectively identify the brain changes associated with each of the classes, we investigate fine tuning framework for classification of AD images based on seven binary classes.
3. To avoid over fitting and be able to generalize the data and reduce validation loss, dropout of 0.2 is introduced to the custom layer over fully connected layer to predict the best result on binary classification.

The following sections make up the remainder of this paper: a literature review about using deep learning (DL) in fMRI is stated in Section 2. Section 3 outlines the proposed approach in detail and is divided into 6 subsections, the first of which is Section 3.1, which describes data that was used in the procedure for evaluating. In Section 3.2, a description of DL for finetuning and classification is discussed. Section 3.3 gives the detailed preprocessing steps while the description of CNN architecture is presented in Section 3.4. In Section 3.5, the description of our proposed fine-tuning model using ResNet18 is explained, and Section 3.6 gives the evaluation measures used to assess the proposed model. The experimental findings are summarized in Section 4, and the discussion is presented in Section 5. The comparison of the proposed model, with existing studies, is presented in Section 6. The paper concludes in Section 7 with the discussion on future research.

## 2. Related Work

The DL algorithms for extracting latent features of neuroimaging data, for early detection of Alzheimer’s disease, have piqued the interest of researchers. To distinguish an Alzheimer’s disease affected brain from a normal (healthy) brain, authors in [19] used CNN to successfully identify functional MRI data of Alzheimer’s patients from standard controls. The model achieved an accuracy of 96.85%. However, more complicated network architecture is required to handle complicated problems. Centered on graph theory and machine learning (ML), authors in [20] created a novel framework for the classification of MCI. The areas of the brain that changed significantly in the MCI groups were correctly described. The proposed model only showed the progression of MCI, differentiating the intermediate stages of the MCI was not considered. Authors in [21] suggested a machine learning-based computer-assisted diagnostic approach that can automatically differentiate Alzheimer’s patients from safe controls. Although the proposed model gave a high prediction accuracy bur it is tough to say exactly which components had an impact on the overall neural network decision. Authors in [22] suggested using fMRI to identify subjects with MCI or AD, incorporating CNN and Ensemble Learning to construct a classifier ensemble (EL). A combined CNN and EL method can find the brain regions that the qualified ensemble model suggests are the most discriminable. The authors concluded that using optimization techniques or other DL approaches, classification accuracy could be improved. To improve the EMCI detection, the Authors in [23] proposed a multi-scale enhanced GCN (MSE-GCN) to investigate individual differences and knowledge association among various subjects. Using image and population phenotypic data, the proposed model was able to learn rich features. With LMCI vs. NC, the accuracy of 93.46% was achieved. More efficient network models for accurate brain region location were suggested by the authors. Authors in [24] proposed a method based on autoencoders for distinguishing between natural aging and disease progression. The proposed approach makes use of effectively biased neural network functionality to accurately diagnose Alzheimer’s disease. Authors in [25] used a 3D CNN to construct a binary classifier that could distinguish between AD and CN resting-state fMRI results. The proposed model used three binary classifications, with AD vs. CN achieving the highest validity accuracy of 97.77%, but there was high computational complexity. Authors in [26] introduced a CNN DL algorithm that predicts who will develop Alzheimer’s disease and who will develop MCI. The proposed model was found to be extremely effective at distinguishing AD and MCI patients from healthy controls, as well as predicting AD conversion. The proposed model did not consider the heterogenous nature of AD. Authors in [27] extracted spatial features from each volume of a 3D static image in an fMRI image sequence. The feature maps were fed into a long short-term memory (LSTM) network to capture the data’s time-varying details. The proposed model had a classification accuracy of 92.11% for AD vs. MCI and 88.12% for MCI vs. NC. However, the multiclass classification accuracy is very low.

For EMCI classification, the authors in [28] proposed a new 3D CNN for removing features that are deeply rooted from dynamic as well as static fMRI brain functional networks with an accuracy of 76.07%. Multi-model, multi-channel, and time-consuming, on the other hand, did not improve classification accuracy. On fMRI, Authors in [29], used a 2D CNN model in conjunction with a transfer learning technique to correctly identify AD, EMCI, and NC. with a 98.41% accuracy. Although the proposed method performed well, it did not deal with the issue of EMCI vs. NC binary classification. Authors in [30] have used a hybrid ML method that utilized bidirectional long short term memory (LSTM) network for identifying discriminative features among AD binary classification from multimodal neuroimaging data. The proposed model had a high run-time complexity. Authors in [31] further develop a novel unified CNN framework using 3D CNN. A 3D Convolutional LSTM (CLSTM) is then applied to extract features and efficiently classified AD binary classes. The proposed model gave an improved classification accuracy, but the Prodromal stages of AD were not considered. Authors in [32] also proposed using CNN fMRI data for early AD classification. Authors in [33] also presented CNN architecture to diagnose AD early using fMRI with an accuracy of 96.7%, but the power of the method to diagnose the disease severity is low. Similarly, the authors in [34] used 3D-CNN on MRI images to obtain high-level features for AD binary classification task with 87.2% accuracy for AD/CN. Authors in [35] further utilized VoxCNN and ResNet for early AD diagnosis and had an accuracy of 80% for AD vs. CN classification. Authors in [36] presented a simple 3D CNN framework, based on the transfer learning strategy for MCI classification, with an accuracy of 94.1%. The proposed model gave a low binary classification accuracy when compared to existing methods.

Furthermore, for six classification tasks, the authors in [37] proposed a layer-wise transfer learning method using VGG 19. The experiments were conducted on 300 ADNI subjects who were divided into six binary groups. With an accuracy of 98.73% on AD vs. NC and 83.72% on EMCI vs. LMCI, the proposed model obtained the best performance but gave a high computational complexity. Authors in [38] further utilized VGG 16 on fMRI dataset for two binary classification tasks. The proposed model was effective in achieving a classification accuracy of 99.27% for AD vs. MCI. The authors recommended other pre-trained networks, such as the Inception Network and the Residual Network for building a better classifier for binary AD classification. Authors in [39] suggested a CNN-based technique for extracting discriminative features from structural MRI with the goal of diagnosing EMCI and LMCI, as well as classifying these two groups from healthy people. Authors in [35] suggested two distinct 3D convolutional network topologies for brain MRI classification and demonstrated the performance of the suggested methodology on the ADNI for the classification of Alzheimer’s disease vs moderate cognitive impairment. Authors in [40] used multimodal data for AD stage classification. Stacked denoising auto-encoders extracted features from genetic and clinical records, whereas 3D CNNs analyzed MRI data to recognize AD vs MCI and healthy controls. Classification of different stages of AD was performed on fMRI dataset, authors in [41] used the architecture of a CNN AlexNet for efficient classification of AD with 97.64% average accuracy. The authors concluded that the use of other pre-trained models, and transfer learning, could improve classification accuracy. Authors in [42] presented an approach for early detection of AD by fine-tuning CaffeNet and GoogLeNet models on 2D MRI images. On an fMRI dataset with AD and NC groups, authors in [43] investigated the performance of ResNet18 based on transfer learning for AD detection. Experiments reported that the proposed model had a 96.88% accuracy. Authors in [44] examined the usefulness of rs-fMRI for multi-class classification of AD and its stages. The classification task was performed using residual neural networks, and the findings showed a wide variety of outcomes depending on the stage of the disease.

The summary of some of the related work that applied DL algorithms on fMRI for early detection of AD is presented in Table 1.

The existing studies suffered from some serious limitations such as low classification accuracy for MCI intermediate classes and non-consideration of binary classes such as EMCI vs. LMCI, EMCI vs. NC. However, there is still a need for more efficient network models for accurate brain region location to aid early detection of AD [23]. Other CNN pre-trained models and more recent cutting-edge networks should be explored as the base model to build an efficient classifier for AD classification [37].

## 3. Methodology

**Figure 1:**
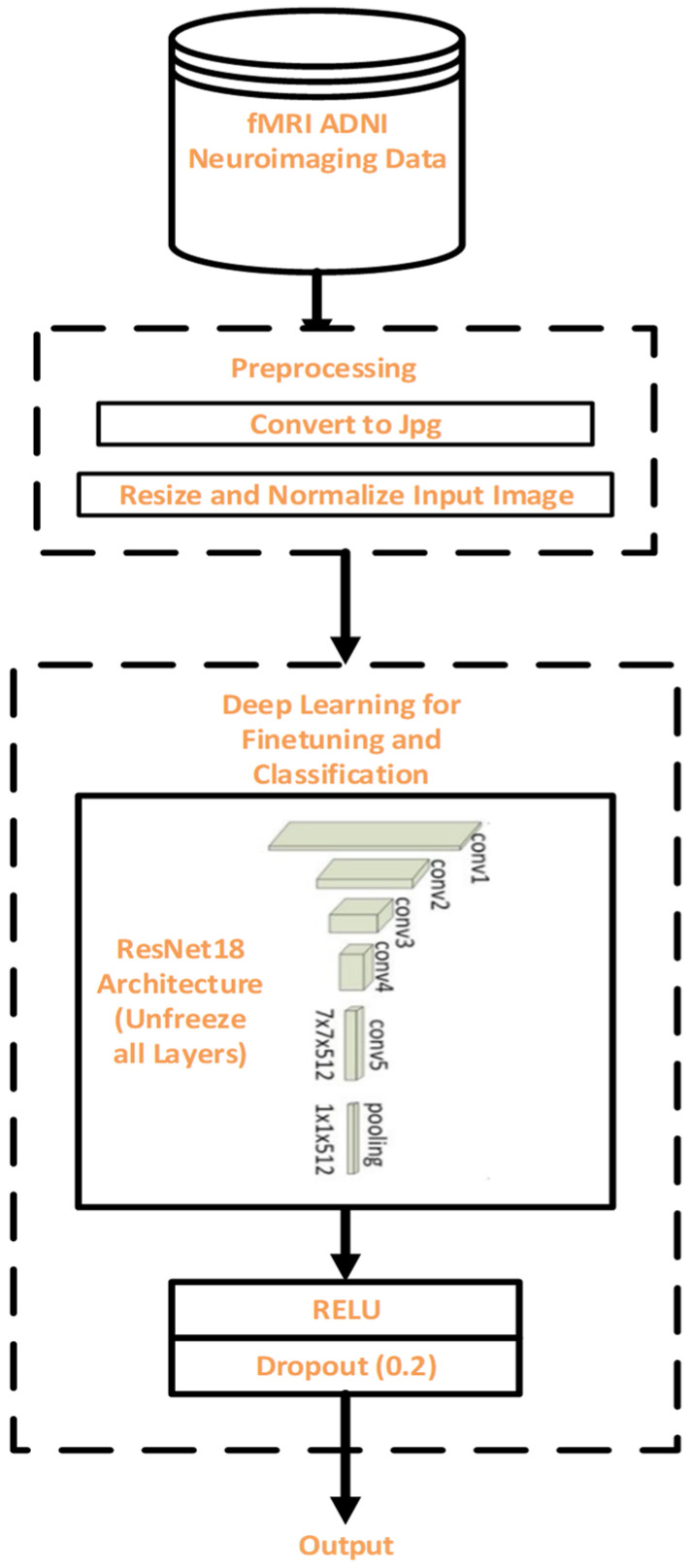
Flow diagram for the proposed methodology.

The research methodology includes data collection, pre-processing, DL-based finetuning and classification, as well as evaluation. A well-known AD database provided the fMRI data. Figure 1 shows the flow diagram of the proposed model.

### 3.1. fMRI Dataset

The study’s data came from the ADNI (Alzheimer’s Disease Neuroimaging Initiative) database (http://adni.loni.usc.edu/ (accessed on January 2021)). There were 413 subjects of six categories with transversal slice orientation resting-state fMR brain imaging in ADNI2 used in this study. For each of the subjects, there is a T1-weighted fMRI image with an axial view in a DICOMM file format. The demographic information related to six categories such as normal control (NC), Mild Cognitive Impairment (MCI), Early MCI (EMCI), Late MCI (LMCI), Significant Memory Concern (SMC), and Alzheimer’s Dementia (AD) is depicted in Table 2. Each subject provided at least 6720 slices from the ADNI database, and slices that prominently show functional properties of the brain region are selected, and 51,443 and 27,310 images were selected for training and validation.

### 3.2. Pre-Processing

The preprocessed ADNI fMRI images are converted from the DICOM (digital imaging and communications in medicine) format to the JPG format. Data enhancement including random resize and cropping to 256 × 256, random rotation, random horizontal flip, center cropping to 224 × 224, conversion to PyTorch tensor, and normalization, based on normalization values for ImageNet [45,46], is performed before inputting the built dataset into the model.

### 3.3. DL Model for Finetuning and Classification

The process of fine-tuning a network is based on the principle of transfer learning. Starting with a pre-trained model, fine-tuning involves training the network on a new dataset and updating all of the model’s parameters. This approach is based on a collection of complicated algorithms that can extract high-level data features from the learn features during training for a broad domain, and a classification function is targeted at minimizing error in that domain. The classification function is further replaced to minimize error on target dataset. Then, the deep neural network contains several parameters (weights) that will be updated during training, thereby transferring its knowledge from the ImageNet dataset to the domain dataset to classify the data with high accuracy.

### 3.4. Proposed Finetuning Model Using ResNet-18 Architecture

ResNet stands for Residual Network, which is an 18-layer CNN proposed by [47]. The ResNet -18 we are using in this study, uses 3 × 3 filters with stride and pad of 1, and the average pooling layer contains 1 × 1 filter, and one fully connected layer, with a final softmax layer. The proposed model is developed by unfreezing all layers, this enables all the parameters of the pre-trained model to adapt to our new dataset.

**Figure 2:**
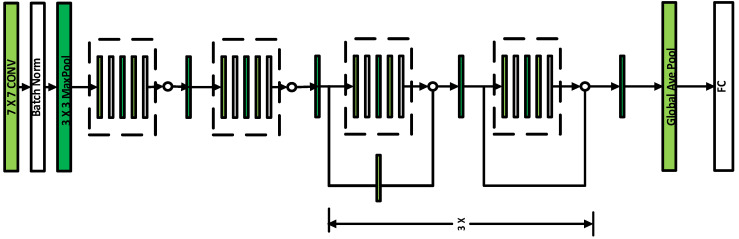
The ResNet-18 Architecture.

The original architecture of ResNet 18 shown in Figure 2, has a total of 17 convolutional layers and one fully connected layer. According to the number of output classes in our dilemma, we change the fully connected layers and reshape the output. First, we perform training from scratch, using the pretrained ResNet18, by unfreezing all the layers, thereby updating all the network parameters. Second, the final dense layer is reshaped to have the same number of inputs as before and to have the same number of outputs as classes in the dataset.

As the performance of CNN depends on the optimality of its parameter values [48], we finally add ReLU and Dropout of 0.2 to build a custom classifier for the classification process. We adapted the non-linear ReLU activation function as it is faster than other non-linear activation functions, and helps to lessen the state of vanishing and error gradient issues [49]. Dropout was varied from 0.1 to 0.4 and 0.2 gave the best performance. Smaller batch size was utilized for the experiment because of the smaller memory of the GPU.

The adapted architecture in our experiment, showing the number of layer parameters, is depicted in Table 3. The hyperparameters for the proposed model used during training and validation is shown in Table 4.

### 3.5. Robustness of the Proposed Model on Various Adverserial Attacks

The abundance of labelled training data are required in most deep learning applications for healthcare. Different adversary attacks occur at various stages of the model development [50]. Some major adversary attacks affecting healthcare applications are poisoning attacks and evasion attacks. Manipulation of training data (poisoning attack) could mislead the training of the deep learning model. The evasion attacks, caused during model inference, could compromise the integrity of the model. In order to avoid some of the adversary attacks in the proposed model, robust features were developed by exploiting connections between different properties of the data. The proposed model was also modified by introducing regularization technique. However, in order to ensure the integrity and authenticity of brain MRI images in telemedicine, robust reversible watermarking [51] should be used to provide copyright protection.

### 3.6. Evaluation Measures

In this study, we assessed the proposed model’s efficiency using a variety of metrics: accuracy, specificity, sensitivity, precision, recall, and f1-score, which is defined in relation to true negative (TN), false negative (FN), true positive (TP), and false positive (FP).

## 4. Results

This section describes the studies that were carried out and addresses the outcomes. We trained two ResNet18 networks (with dropout and without dropout) to perform seven binary classifications, including NC vs. AD, NC vs. EMCI, NC vs. LMCI, EMCI vs. LMCI, EMCI vs. AD, LMCI vs. AD, and MCI vs. EMCI. The dataset consists of fMRI of 138 subjects with a total of 78,753 images. For the evaluation, we split the dataset into the training dataset and validation dataset with 70% (17 subjects consisting of 51,443 images) and 30% (8 subjects consisting of 27,310 images) split ratio respectively as described in Table 5.

### 4.1. Result Based on ResNet18 without Dropout

In this study, we first evaluated the modified ResNet18, without Dropout, on the seven binary classification scenarios and established the results on validation data using the hyperparameters depicted in Table 3. Furthermore, we explored reducing overfitting using early stopping to optimize the epoch’s size hyperparameter. Our model without dropout achieved validation accuracy result 99.45%, 96.51%, and 99.9% on EMCI vs. LMCI, CN vs. EMCI, EMCI vs. AD classification respectively as shown in Table 6.

### 4.2. Result Based on ResNet18 with Dropout

**Figure 3:**
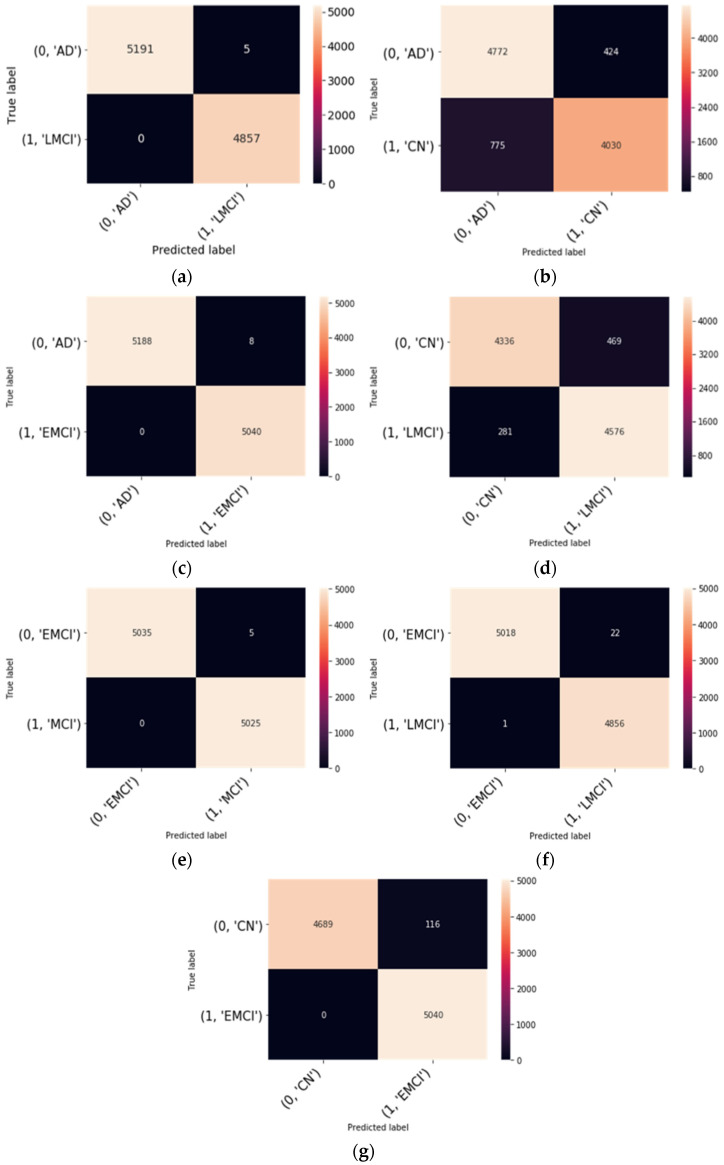
Confusion matrix of the proposed model for binary classification: (**a**) LMCI vs. AD; (**b**) CN vs. AD; (**c**), AD vs. EMCI (**d**), CN vs. LMCI (**e**), EMCI vs. MCI (**f**), EMCI vs. LMCI (**g**), CN vs. EMCI.

This subsection discussed the result obtained from our proposed finetuning model as depicted in Figure 1. The PyTorch library was used with Python in all of the experiments. For our proposed model, we used the hyperparameters depicted in Table 4. The architecture of our modified ResNet18 is shown in Table 2. To ascertain the effectiveness of our modified ResNet18 on the ADNI dataset, the average values of accuracy, sensitivity, and specificity are depicted in Table 7. In addition, the confusion matrices of the modified ResNet18 model on the seven binary classes were also computed to explain the performance of classification on the validation data. Figure 3 depicts the confusion matrices. We also measured performance metrics, such as precision, recall, and f1-score, using the confusion matrices. The overall classification performance of the proposed model on all the seven binary classification scenarios is shown in Table 8.

## 5. Discussion

In this study, we have analyzed the effect of dropout on a fine-tuned pretrained model to classify fMR images from the ADNI database. This study’s findings revealed that fine-tuning the entire network gave high classification accuracy on all binary classification scenarios except AD vs. CN and CN vs. LMCI. Without dropout the best performance was achieved by EMCI vs. AD with an accuracy of 99.99% (Table 7). Table 8 shows the effect of dropout on the binary classification. We can see that the proposed model has yielded positive results on EMCI vs. AD, EMCI vs. LMCI, LMCI vs. AD, EMCI vs. MCI classification and achieved 99.99%, 99.76%, 99.95%, and 99.95% accuracy respectively. In terms of sensitivity, however, the AD vs. CN classification performance was superior. in the case of the model, without dropout, with a value of 97.8%. Regarding the confusion matrices in Figure 3, no subjects are misdiagnosed as AD as seen from the binary classification of AD vs. LMCI and AD vs. EMCI as shown in Figure 3. Likewise, no subjects are misdiagnosed as EMCI and CN, respectively. However, few subjects are misdiagnosed as EMCI as in the case of EMCI vs. LMCI. This suggests that the proposed model is feasible and can correctly classify the intermediate stages of MCI, and using useful features derived from functional brain networks, the proposed model could effectively differentiate EMCI from LMCI.

With the high classification accuracy performance, the fine-tuning model produced some overfitting. To elucidate the overfitting hurdle on a noisy dataset that causes the model to learn patterns from the training data that do not generalize to the validation data, regularization technique such as dropout plays a major role. We observed that the dropout does not help in alleviating the overfitting. This finding indicates that the proposed model recognized the pattern in differentiating between the intermediary stages of MCI with the regularization technique. This corroborates the idea that the proposed network gave high precision in most of the binary classification, as shown in Table 8. The use of the regularization technique for training allowed for the obtaining of better models, thus increasing the classification accuracy.

## 6. Comparison with Existing Studies

To validate our proposed approach, we compared our findings to previous studies that investigated the early diagnosis of AD using binary classification, as shown in Table 9, Table 10 and Table 11. The proposed model gives better result in terms of accuracy, sensitivity, and specificity with 98.74% accuracy, 97.24% sensitivity, and 100% specificity on CN vs. EMCI classification scenario, and 99.76% accuracy, 99.56% sensitivity, and 99.97% specificity on EMCI vs. LMCI classification scenario.

**Figure 4:**
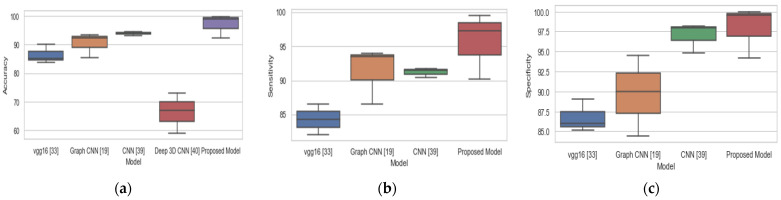
Comparison of proposed method and existing methods for binary classification: (**a**) accuracy, (**b**) sensitivity, (**c**) specificity.

The study [23] achieved 93.46%, 94.03%, and 92.50% in terms of accuracy, sensitivity, and specificity respectively for CN vs. LMCI binary classification and thereby outperformed our proposed method in terms of accuracy and sensitivity. Likewise, the study [39] outperformed our proposed model in all the three-performance metrics for CN vs LMCI. The overall performance of our proposed model on the three binary classification tasks is based on accuracy, sensitivity, and specificity, and this is compared with existing methods as represented with box plots in Figure 4. Our proposed model achieved the best performance with a median accuracy of 98.9%, median sensitivity of 98%, and median specificity of 99.9% over three binary classification tasks. Our proposed model achieved the highest accuracy, sensitivity, and specificity of 98.74%, 97.24%, and 100%, respectively, for CN vs EMCI binary classification as compared to other existing methods. In LMCI vs EMCI binary classification, our proposed method achieved a highest accuracy, sensitivity, and specificity of 99.76%, 99.56%, and 99.97%, respectively. By comparing the findings of this study to the findings of other research, we may conclude that our proposed system is a more trustworthy and accurate method.

For the clinical applicability of the proposed model in diseases such as stroke, most stroke survivors suffered from some cognitive functions, such as such as attention, concentration, memory, social cognition, language, spatial, and perceptual skills. The cognitive impairments for stroke survivors have not been addressed adequately. However, findings show that patients with neurocognitive disorders caused by AD had a higher level of affective suffering than those with neurocognitive disorders caused by stroke [52].

## 7. Conclusions

AD is a debilitating brain disease that cannot be cured, and it impacts a large portion of the aging world’s population. The need to diagnose this disease early to establish effective care and enhance patients’ lives cannot be over-emphasized. This study proposed a modified ResNet18 fine-tuning approach for accurately classifying fMRI brain slices among seven binary classification tasks: CN vs. AD, CN vs. EMCI, CN vs. LMCI, EMCI vs. LMCI, EMCI vs. AD, LMCI vs. AD, and EMCI vs. MCI. The training data contained information about 61,502 images and the validation samples contained 30,095 samples. This study was able to address the problem of overfitting by finetuning all the convolutional layers and regularizing using a dropout of 0.2. This paper investigated the performance of two deep learning models (ResNet18 model without dropout and ResNet18 model with dropout) on the seven binary classification tasks. We demonstrated that finetuning ResNet18, and training it from scratch, was able to extract meaningful features for the seven binary classification tasks. The analysis results for our proposed model shows that, for regularizing with 0.2 dropout, the model was able to effectively diagnose AD early without any false positive but with very low false negative on the seven binary classification tasks. Our model achieved the best classification accuracy of 99.99%, 99.95%, and 99.95% for EMCI vs. AD, LMCI vs. AD, and MCI vs. EMCI, respectively. Additionally, for the proposed model, for EMCI vs. AD, LMCI vs. AD, and MCI vs. EMCI, the sensitivity is 99.84%, 99.90%, and 99.90%, respectively. The comparison of our method with existing methods shows that finetuning with regularization not only reduced overfitting but was also able to improve classification accuracy with low misclassification error.

For ascertaining and explaining the model decision, the use of visualization techniques (such as based on the neural network activations) will be considered in the future. We will also investigate other, recently proposed, neural network models for building classifiers in future studies. We will also explore a hybrid model, based on the pre-trained CNN, to achieve a better classification model with fewer false negatives. The performance of the model on a multiclass classification case also will be investigated in the future.

## Figures and Tables

**Table 1:** Summary of Related Works.

References	Deep Learning Model	Brain Region	Contribution	Limitations
[30]	VGG 19	Gray Matter	Model provided clear distinction between EMCI and LMC	Low classification accuracy for intermediate stages
[22]	CNN with Ensemble Learning	Whole brain	Baseline	Low classification accuracy for MCIc vs. MCI nc
[31]	3D Convolutional LSTM	Whole brain	Accuracy was improved	Prodromal stages of AD were not considered
[25]	3D CNN	Whole brain	Model learned temporal and spatial features	Model was designed for a specific size of fMRI volume
[28]	CNN with residual connection	Hippocampus	Baseline	N/A
[29]	Inception-ResNet V2	Whole brain	Based on various age groups, the model was able to extrapolate the prediction of different phases	Model did not address the problem of EMCI vs. NC binary classification
[33]	CNN	Whole brain	Model recognized pattern of brain functional changes associated with AD progression	Intermediate stages of AD were not considered
[36]	AlexNet	Whole brain	Model classified all stages of AD	Low binary classification accuracy
[37]	VGG16	Whole brain	Model was able to extract useful features the binary classification tasks	High computational complexity

**Table 2:** The dataset’s demographic data.

	CN	MCI	EMCI	LMCI	SMC	AD
No. of Subjects	25	13	25	25	25	25
Sex (M/F)	10/15	7/6	13/12	10/15	10/15	10/15
Mean Age (years)	76.42	75.64	69.84	73.11	71.37	75.34

**Table 3:** The modified ResNet18 Architecture for AD Classification.

Layer Type	No of Parameters
Conv2d-1	9408
BatchNorm2d-2	128
Conv2d-5	36,864
BatchNorm2d-6	128
Conv2d-8	36,864
BatchNorm2d-9	128
Conv2d-12	36,864
BatchNorm2d-13	128
Conv2d-15	36,864
BatchNorm2d-16	128
Conv2d-19	73,728
BatchNorm2d-20	256
Conv2d-22	147,456
BatchNorm2d-23	256
Conv2d-24	8192
BatchNorm2d-25	256
Conv2d-28	147,456
BatchNorm2d-29	256
Conv2d-31	147,456
BatchNorm2d-32	256
Conv2d-35	294,912
BatchNorm2d-36	512
Conv2d-38	589,824
BatchNorm2d-39	512
Conv2d-40	32,768
BatchNorm2d-41	512
Conv2d-44	589,824
BatchNorm2d-45	512
Conv2d-47	589,824
BatchNorm2d-48	512
Conv2d-51	1,179,648
BatchNorm2d-52	1024
Conv2d-54	2,359,296
BatchNorm2d-55	1024
Conv2d-56	131,072
BatchNorm2d-57	1024
Conv2d-60	2,359,296
BatchNorm2d-61	1024
Conv2d-63	2,359,296
BatchNorm2d-64	1024
Linear-68	131,328
Dropout-70	0
Linear-71	1542
LogSoftmax-72	0

**Table 4:** Hyperparameters for the Proposed Model.

Hyperparameters	Name/Value
Function (activation)	ReLU
Rate of learning	1 × 10^−5^
Epochs	20 with early stopping
Batch size	4
Loss function	Cross Entropy
Optimizer	Stochastic Gradient Descent (SGD)

**Table 5:** Details of the dataset split for training and validation.

Class Name	Training Dataset (70%)	Validation Dataset (30%)
CN	10,523	4805
LMCI	10,421	4857
AD	10,264	5196
SMC	10,155	5192
EMCI	10,080	5040
MCI	10,059	5025

**Table 6:** Evaluation Metric on Validation Data for Model without Dropout.

Binary Classes	Accuracy (%)	Sensitivity (%)	Specificity (%)
EMCI vs. LMCI	99.45	100	98.88
AD vs. CN	75.12	97.8	50.59
CN vs/EMCI	96.51	98.62	99.96
CN vs. LMCI	74.91	67.36	97.92
EMCI vs. AD	99.90	100	99.70
LMCI vs. AD	99.34	98.72	100
MCI vs. EMCI	99.98	99.96	100

**Table 7:** Metrics of Evaluation on Validation Data for Model with Dropout.

Binary Classes	Accuracy (%)	Sensitivity (%)	Specificity (%)
EMCI vs. LMCI	99.76	99.56	99.97
AD vs. CN	80.80	91.83	83.87
CN vs/EMCI	98.74	97.24	100
CN vs. LMCI	92.23	90.23	94.21
EMCI vs. AD	99.99	99.84	100
LMCI vs. AD	99.95	99.90	100
MCI vs. EMCI	99.95	99.90	100

**Table 8:** Precision, recall, and f1-score for seven binary classification tasks.

Binary Classes	Class Label	Precision	Recall	F1-Score
EMCI vs. LMCI	EMCI	1.00	0.99	0.99
	LMCI	0.99	1.00	0.99
AD vs. CN	AD	0.86	0.92	0.89
	CN	0.90	0.84	0.87
CN vs/EMCI	CN	1.00	0.98	0.99
	EMCI	0.98	1.00	0.99
CN vs. LMCI	CN	0.94	0.90	0.92
	LMCI	0.91	0.94	0.92
EMCI vs. AD	AD	1.00	1.00	1.00
	EMCI	1.00	1.00	1.00
LMCI vs. AD	AD	1.00	1.00	1.00
	LMCI	1.00	1.00	1.00
MCI vs. EMCI	EMCI	1.00	1.00	1.00
	MCI	1.00	1.00	1.00

**Table 9:** Evaluation Results with the CN vs. EMCI Binary Classification.

Reference	Methodology	Accuracy (%)	Sensitivity (%)	Specificity (%)
[37]	Vgg16	85.16	84.29	85.98
[23]	Graph CNN	85.42	86.57	84.42
[39]	CNN	93.96	90.46	98.19
[35]	Deep 3D CNN	59	-	-
Proposed Model	Modified ResNet18	98.74	97.24	100

**Table 10:** Evaluation Results with the CN vs. LMCI Binary Classification.

Reference	Methodology	Accuracy (%)	Sensitivity (%)	Specificity (%)
[37]	Vgg16	89.91	86.61	89.01
[23]	Graph CNN	93.46	94.03	92.50
[39]	CNN	94.54	91.70	97.96
[35]	Deep 3D CNN	73	-	-
Proposed Model	Modified ResNet18	92.23	90.23	94.21

**Table 11:** Evaluation Results with the EMCI vs. LMCI Binary Classification.

Reference	Methodology	Accuracy (%)	Sensitivity (%)	Specificity (%)
[37]	Vgg16	83.72	82.09	85.13
[23]	Graph CNN	92.31	93.51	90.00
[39]	CNN	93.00	91.48	94.82
[35]	Deep 3D CNN	67	-	-
Proposed Model	Modified ResNet18	99.76	99.56	99.97

## Data Availability

Data is available from the corresponding author upon reasonable request.

## References

[1] Ding Y., Sohn J.H., Kawczynski M.G., Trivedi H., Harnish R., Jenkins N.W., Lituiev D., Copeland T.P., Aboian M.S., Aparici C.M. (2019). A Deep Learning Model to Predict a Diagnosis of Alzheimer Disease by Using 18F-FDG PET of the Brain. Radiology.

[2] Zhang F., Li Z., Zhang B., Du H., Wang B., Zhang X. (2019). Multi-modal deep learning model for auxiliary diagnosis of Alzheimer’s disease. Neurocomputing.

[3] Wang Y., Xu C., Park J.-H., Lee S., Stern Y., Yoo S., Kim J.H., Kim H.S., Cha J. (2019). Diagnosis and prognosis of Alzheimer’s disease using brain morphometry and white matter connectomes. NeuroImage Clin..

[4] Alberdi A., Weakley A., Schmitter-Edgecombe M., Cook D.J., Aztiria A., Basarab A., Barrenechea M. (2018). Smart Home-Based Prediction of Multidomain Symptoms Related to Alzheimer’s Disease. IEEE J. Biomed. Health Inform..

[5] Li H., Habes M., Wolk D.A., Fan Y. (2019). A deep learning model for early prediction of Alzheimer’s disease dementia based on hippocampal MRI. arXiv.

[6] Pavisic I.M., Pertzov Y., Nicholas J.M., O’Connor A., Lu K., Yong K.X.X., Husain M., Fox N.C., Crutch S.J. (2021). Eye-tracking indices of impaired encoding of visual short-term memory in familial Alzheimer’s disease. Sci. Rep..

[7] López-De-Ipiña K., Martinez-De-Lizarduy U., Calvo P.M., Beitia B., García-Melero J., Fernández E., Ecay-Torres M., Faundez-Zanuy M., Sanz P. (2020). On the analysis of speech and disfluencies for automatic detection of Mild Cognitive Impairment. Neural Comput. Appl..

[8] Sapey-Triomphe L.-A., Heckemann R.A., Boublay N., Dorey J.-M., Hénaff M.-A., Rouch I., Padovan C., Hammers A., Krolak-Salmon P. (2015). Neuroanatomical Correlates of Recognizing Face Expressions in Mild Stages of Alzheimer’s Disease. PLoS ONE.

[9] Tzimourta K.D., Afrantou T., Ioannidis P., Karatzikou M., Tzallas A.T., Giannakeas N., Astrakas L.G., Angelidis P., Glavas E., Grigoriadis N. (2019). Analysis of electroencephalographic signals complexity regarding Alzheimer’s Disease. Comput. Electr. Eng..

[10] Krishna N.M., Sekaran K., Vamsi A.V.N., Ghantasala G.S.P., Chandana P., Kadry S., Blazauskas T., Damasevicius R., Kaushik S. (2019). An Efficient Mixture Model Approach in Brain-Machine Interface Systems for Extracting the Psychological Status of Mentally Impaired Persons Using EEG Signals. IEEE Access.

[11] Chandra A., Dervenoulas G., Politis M. (2019). Magnetic resonance imaging in Alzheimer’s disease and mild cognitive impairment. J. Neurol..

[12] Ke Q., Zhang J., Wei W., Damasevicius R., Wozniak M. (2019). Adaptive Independent Subspace Analysis of Brain Magnetic Resonance Imaging Data. IEEE Access.

[13] Zheng Y., Guo H., Zhang L., Wu J., Li Q., Lv F. (2019). Machine Learning-Based Framework for Differential Diagnosis Between Vascular Dementia and Alzheimer’s Disease Using Structural MRI Features. Front. Neurol..

[14] Lian C., Liu M., Zhang J., Shen D. (2020). Hierarchical Fully Convolutional Network for Joint Atrophy Localization and Alzheimer’s Disease Diagnosis Using Structural MRI. IEEE Trans. Pattern Anal. Mach. Intell..

[15] Maggipinto T., Bellotti R., Amoroso N., Diacono D., Donvito G., Lella E., Monaco A., Scelsi M., Tangaro S. (2017). DTI measurements for Alzheimer’s classification. Phys. Med. Biol..

[16] Chitradevi D., Prabha S. (2020). Analysis of brain sub regions using optimization techniques and deep learning method in Alzheimer disease. Appl. Soft Comput..

[17] Lu D., Popuri K., Ding G.W., Balachandar R., Beg M.F. (2018). Multimodal and Multiscale Deep Neural Networks for the Early Diagnosis of Alzheimer’s Disease using structural MR and FDG-PET images. Sci. Rep..

[18] Gorges M., Müller H.-P., Kassubek J. (2018). Structural and Functional Brain Mapping Correlates of Impaired Eye Movement Control in Parkinsonian Syndromes: A Systems-Based Concept. Front. Neurol..

[19] Sarraf S., Tofighi G. (2016). Classification of Alzheimer’s Disease using fMRI Data and Deep Learning Convolutional Neural Networks. arXiv.

[20] Hojjati S.H., Ebrahimzadeh A., Khazaee A., Babajani-Feremi A. (2017). Predicting conversion from MCI to AD using resting-state fMRI, graph theoretical approach and SVM. J. Neurosci. Methods.

[21] Duc N.T., Ryu S., Qureshi M.N.I., Choi M., Lee K.H., Lee B. (2019). 3D-Deep Learning Based Automatic Diagnosis of Alzheimer’s Disease with Joint MMSE Prediction Using Resting-State fMRI. Neuroinformatics.

[22] Pan D., Zeng A., Jia L., Huang Y., Frizzell T., Song X. (2020). Early Detection of Alzheimer’s Disease Using Magnetic Resonance Imaging: A Novel Approach Combining Convolutional Neural Networks and Ensemble Learning. Front. Neurosci..

[23] Yu S., Wang S., Xiao X., Cao J., Yue G., Liu D., Wang T., Xu Y., Lei B. (2020). Multi-scale Enhanced Graph Convolutional Network for Early Mild Cognitive Impairment Detection. Medical Image Computing and Computer Assisted Intervention—MICCAI 2020.

[24] Guo H., Zhang Y. (2020). Resting State fMRI and Improved Deep Learning Algorithm for Earlier Detection of Alzheimer’s Disease. IEEE Access.

[25] Parmar H., Nutter B., Long R., Antani S., Mitra S. (2020). Spatiotemporal feature extraction and classification of Alzheimer’s disease using deep learning 3D-CNN for fMRI data. J. Med Imaging.

[26] Basaia S., Agosta F., Wagner L., Canu E., Magnani G., Santangelo R., Filippi M. (2019). Automated classification of Alzheimer’s disease and mild cognitive impairment using a single MRI and deep neural networks. NeuroImage Clin..

[27] Li W., Lin X., Chen X. (2020). Detecting Alzheimer’s disease Based on 4D fMRI: An exploration under deep learning framework. Neurocomputing.

[28] Kam T.-E., Zhang H., Shen D. (2018). A Novel Deep Learning Framework on Brain Functional Networks for Early MCI Diagnosis. Medical Image Computing and Computer Assisted Intervention—MICCAI 2018.

[29] Puranik M., Shah H., Shah K., Bagul S. (2018). Intelligent Alzheimer’s Detector Using Deep Learning. Proceedings of the 2nd International Conference on Intelligent Computing and Control Systems (ICICCS).

[30] Abuhmed T., El-Sappagh S., Alonso J.M. (2021). Robust hybrid deep learning models for Alzheimer’s progression detection. Knowledge-Based Syst..

[31] Xia Z., Yue G., Xu Y., Feng C., Yang M., Wang T., Lei B. A Novel End-to-End Hybrid Network for Alzheimer’s Disease Detection Using 3D CNN and 3D CLSTM. Proceedings of the 2020 IEEE 17th International Symposium on Biomedical Imaging (ISBI).

[32] Khanna A., Tanwar S., Rodrigues J.J., Roy N.R. (2019). Alzheimer detection using Group Grey Wolf Optimization based features with convolutional classifier. Comput. Electr. Eng..

[33] Amini M., Pedram M., Moradi A., Ouchani M. (2021). Diagnosis of Alzheimer’s Disease Severity with fMRI Images Using Robust Multitask Feature Extraction Method and Convolutional Neural Network (CNN). Comput. Math. Methods Med..

[34] Cheng D., Liu M., Fu J., Wang Y. Classification of MR brain images by combination of multi-CNNs for AD diagnosis. Proceedings of the 9th International Conference on Digital Image Processing (ICDIP 2017).

[35] Korolev S., Safiullin A., Belyaev M., Dodonova Y. Residual and plain convolutional neural networks for 3D brain MRI classification. Proceedings of the 2017 IEEE 14th International Symposium on Biomedical Imaging (ISBI 2017).

[36] Esmaeilzadeh S., Belivanis D.I., Pohl K.M., Adeli E. (2018). End-To-End Alzheimer’s Disease Diagnosis and Biomarker Identification. Machine Learning in Medical Imaging.

[37] Mehmood A., Yang S., Feng Z., Wang M., Ahmad A.S., Khan R., Maqsood M., Yaqub M. (2021). A Transfer Learning Approach for Early Diagnosis of Alzheimer’s Disease on MRI Images. Neuroscience.

[38] Jain R., Jain N., Aggarwal A., Hemanth D.J. (2019). Convolutional neural network based Alzheimer’s disease classification from magnetic resonance brain images. Cogn. Syst. Res..

[39] Gorji H.T., Kaabouch N. (2019). A Deep Learning approach for Diagnosis of Mild Cognitive Impairment Based on MRI Images. Brain Sci..

[40] Venugopalan J., Tong L., Hassanzadeh H.R., Wang M.D. (2021). Multimodal deep learning models for early detection of Alzheimer’s disease stage. Sci. Rep..

[41] Kazemi Y., Houghten S. A deep learning pipeline to classify different stages of Alzheimer’s disease from fMRI data. Proceedings of the 2018 IEEE Conference on Computational Intelligence in Bioinformatics and Computational Biology (CIBCB).

[42] Guo S., Xiao B., Wu C. (2020). Identifying subtypes of mild cognitive impairment from healthy aging based on multiple cortical features combined with volumetric measurements of the hippocampal subfields. Quant. Imaging Med. Surg..

[43] Ebrahimi A., Luo S., Chiong R. Introducing Transfer Learning to 3D ResNet-18 for Alzheimer’s Disease Detection on MRI Images. Proceedings of the 2020 35th International Conference on Image and Vision Computing New Zealand (IVCNZ).

[44] Ramzan F., Khan M.U.G., Rehmat A., Iqbal S., Saba T., Rehman A., Mehmood Z. (2020). A Deep Learning Approach for Automated Diagnosis and Multi-Class Classification of Alzheimer’s Disease Stages Using Resting-State fMRI and Residual Neural Networks. J. Med Syst..

[45] Simonyan K., Zisserman A. (2014). Very deep convolutional networks for large-scale image recognition. arXiv.

[46] Krizhevsky A., Sutskever I., Hinton G.E. (2012). Imagenet classification with deep convolutional neural networks. Commun. ACM.

[47] He K., Zhang X., Ren S., Sun J. Deep residual learning for image recognition. Proceedings of the IEEE Conference on Computer Vision and Pattern Recognition.

[48] Vinayakumar R., Soman K.P., Poornachandran P. Applying convolutional neural network for network intrusion detection. Proceedings of the 2017 International Conference on Advances in Computing, Communications and Informatics (ICACCI).

[49] Vinayakumar R., Alazab M., Soman K.P., Poornachandran P., Al-Nemrat A., Venkatraman S. (2019). Deep Learning Approach for Intelligent Intrusion Detection System. IEEE Access.

[50] Ravi V., Alazab M., Srinivasan S., Arunachalam A., Soman K.P. (2021). Adversarial Defense: DGA-Based Botnets and DNS Homographs Detection Through Integrated Deep Learning. IEEE Trans. Eng. Manag..

[51] Zhou X., Ma Y., Zhang Q., Mohammed M., Damaševičius R. (2021). A Reversible Watermarking System for Medical Color Images: Balancing Capacity, Imperceptibility, and Robustness. Electronics.

[52] Dindelegan C.M., Faur D., Purza L., Bumbu A., Sabau M. (2020). Distress in neurocognitive disorders due to Alzheimer’s disease and stroke. Exp. Ther. Med..

